# Endoscope-Assisted Microneurosurgery for Intracranial Aneurysms

**DOI:** 10.3389/fneur.2013.00201

**Published:** 2013-12-18

**Authors:** Renato J. Galzio, Francesco Di Cola, Soheila Raysi Dehcordi, Alessandro Ricci, Danilo De Paulis

**Affiliations:** ^1^Department of Neurosurgery, University of L’Aquila, L’Aquila, Italy; ^2^Department of Neurosurgery, San Salvatore City Hospital, L’Aquila, Italy

**Keywords:** intracranial aneurysms, endoscopic assistance, microneurosurgery, endoscopic instruments, anatomic investigations

## Abstract

**Background:** The endovascular techniques has widely changed the treatment of intracranial aneurysms. However surgery still represent the best therapeutic option in case of broad-based and complex lesions. The combined use of endoscopic and microsurgical techniques (EAM) may improve surgical results.

**Objective:** The purpose of our study is to evaluate the advantages and limits of EAM for intracranial aneurysms.

**Methods:** Between January 2002 and December 2012, 173 patients, harboring 206 aneurysms were surgically treated in our department with the EAM technique. One hundred and fifty-seven aneurysms were located in the anterior circulation and 49 were in the posterior circulation. Standard tailored approaches, based on skull base surgery principles, were chosen. The use of the endoscope included three steps: initial inspection, true operative time, and final inspection. For each procedure, an intraoperative video and an evaluation schedule were prepared, to report surgeons’ opinions about the technique itself. In the first cases, we always used the endoscope during surgical procedures in order to get an adequate surgical training. Afterwards we became aware in selecting cases in which to apply the endoscopy, as we started to become familiar with its advantages and limits.

**Results:** After clipping, all patients were undergone postoperative cerebral angiography. No surgical mortality related to EAM were observed. Complications directly related to endoscopic procedures were rare.

**Conclusion:** Our retrospective study suggests that endoscopic efficacy for aneurysms is only scarcely influenced by the preoperative clinical condition (Hunt–Hess grade), surgical timing, presence of blood in the cisterns (Fisher grade) and/or hydrocephalus. However the most important factors contributing to the efficacy of EAM are determined by the anatomical locations and sizes of the lesions. Furthermore, the advantages are especially evident using dedicated scopes and holders, after an adequate surgical training to increase the learning curve.

## Introduction

The advent of endovascular techniques has widely changed the treatment of intracranial aneurysms. However, surgery still represents the best therapeutic option in case of broad-based and complex lesions. Better outcomes can be obtained only in centers where both treatment options (endovascular and surgical) are carried out by skilled teams. The application of any additional method and technique may improve surgical results. The combined use of endoscopic and microsurgical techniques has been already discussed as a possible methodological improvement in treatment of deeply located lesions (1–5).

Hopf and Perneczky have categorized endoscopy manipulations into Endoscopic neurosurgery (EN), endoscope-assisted microneurosurgery (EAM), and endoscope-controlled microneurosurgery (ECM); EN is used for all exclusively endoscopic procedures. Surgical manipulations are performed with special endoscopic instruments through the endoscope itself. EAM is used for procedures in which endoscopy is performed in addition to microsurgical manipulations under the operating microscope during the same operation (6–8). ECM is used for procedures in which microsurgical manipulations are performed with conventional instruments but are guided by the video image provided by the endoscope (8, 9). The purpose of our study is to evaluate the advantages and the limits of these techniques in intracranial aneurysms surgery.

## Materials and Methods

### Patient population

During a 10-year period (January 2003–December 2012), at the Department of Neurosurgery of the San Salvatore City Hospital of L’Aquila in Italy, we evaluated the clinical history of every single patient harboring intracranial aneurysms, in association with neuroradiologists in order to decide the best possible treatment. For this study, we only selected patients who had undergone surgical treatment. Thereafter, 173 patients harboring 206 aneurysms and treated in 181 consecutive surgical procedures were considered. There were 76 men and 97 women; the age of these patients ranged from 13 to 82 years (mean 49.5). Forty-one patients (23.7%) presented unruptured aneurysms and 132 had a hemorrhagic onset (76.3%). Among patients with ruptured aneurysms, 55 were operated in H–H grade I, 56 in grade II, and 21 in grade III. Eighty-six patients (65.2%) were operated on within 48 h from bleeding, 21 patients (15.9%) were treated within the third and the seventh post-hemorrhagic day and 25 patients (19.9%) were operated later. Hemorrhagic patients with a H–H grade 4 or 5 (Hunt and Hess scale) as well as patients with intracavernous lesions, were excluded. The size of the lesions ranged from 4 to 40 mm. Eighteen patients harbored multiple aneurysms: 13 patients presented two lesions, 3 patients presented three lesions, 1 patient presented four aneurysms and in one case six aneurysms were revealed. Eight patients with multiple aneurysms were submitted to two different surgical procedures, because treatment with the same surgical approach was not appropriate due to the location of the lesions.

Consequently, 157 aneurysms were located in the anterior circulation and the affected sites were: internal carotid artery (ICA) in 62 cases, 16 of which originating from ICA-ophthalmic artery (ICA-Opht artery) in the anterior wall of ICA and 46 originating from ICA-posterior communicating artery (ICA-PcomA) and ICA-anterior choroidal artery (ICA-AchorA) in the posterior wall of ICA, ICA bifurcation in 21 cases, middle cerebral artery (MCA) in 40 cases, anterior cerebral artery/anterior communicating artery (ACA/AcomA) in 33 cases. Forty-nine aneurysms were located in the posterior circulation and the affected sites were: basilar apex (BA) in 13 cases, midbasilar artery (MA) in 4 cases, vertebral-basilar junction (VBJ) in 6 cases, vertebral artery (VA) in 17 cases, and distal arteries in 10 cases (Table 1).

**Table 1:** **Quantity and distribution of the treated aneurysms from January 2002 to December 2012 for a total of 206 aneurysms in 173 patients**.

	Number	%
MCA	40	19.41
ICA-anterior wall (ICA/Opht)	16	7.76
ICA-posterior wall (ICA/AchorA, ICA/PcomA)	46	22.33
ICA bifurcation	21	10.19
ACA/AcomA	33	16.05
BA	13	6.31
MA	4	1.94
VBJ	6	2.91
VA	17	8.25
Distal arteries	10	4.85

*MCA, middle cerebral artery; ICA, internal carotid artery; Opht, ophthalmic; AchorA, anterior choroidal artery; PcomA, posterior communicating artery; ACA, anterior cerebral artery; AcomA, anterior communicating artery; BA, basilar artery; MA, midbasilar artery; VBJ, vertebral-basilar junction; VA, vertebral artery*.

In the first series we always used endoscope in order to achieve adequate surgical training. Afterward when selecting cases, we became aware of those in which to apply the endoscopy, as we became more familiar with its advantages and limits. In any case an intraoperative video and an evaluation schedule were prepared for each procedure, to report surgeons’ opinions about the technique itself.

### Surgical procedure and technique

**Figure 1:**
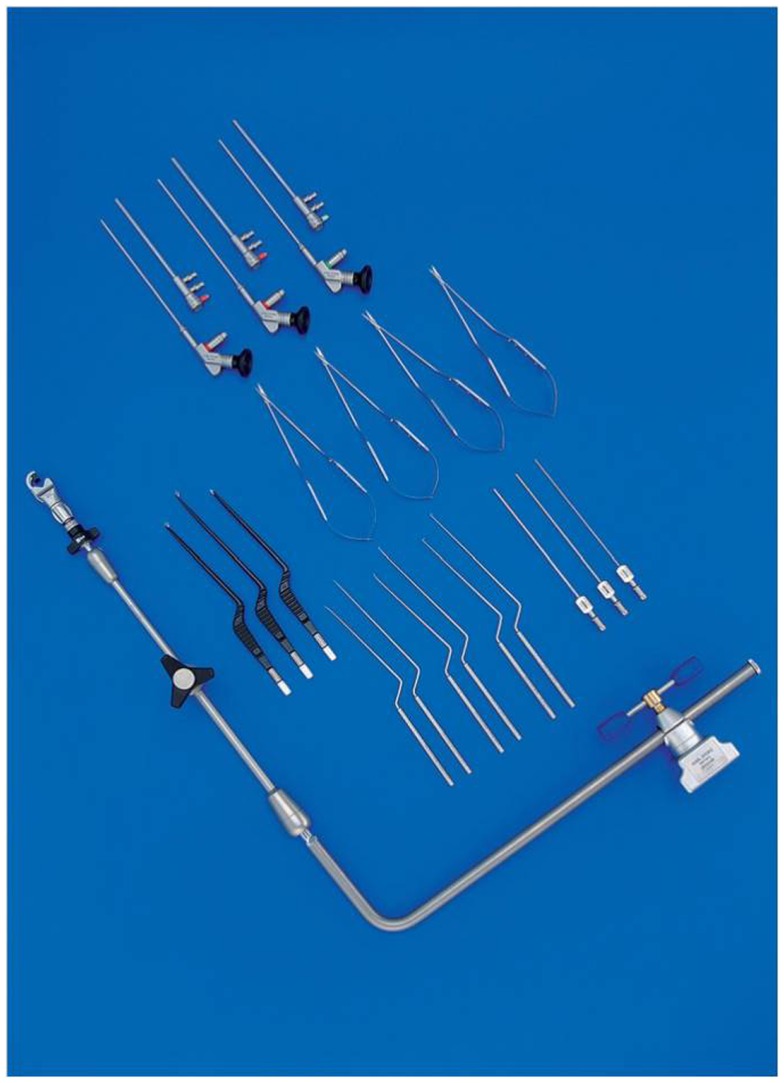
**Endoscopic instruments and mechanical holding system retractor arm used**.

**Figure 2:**
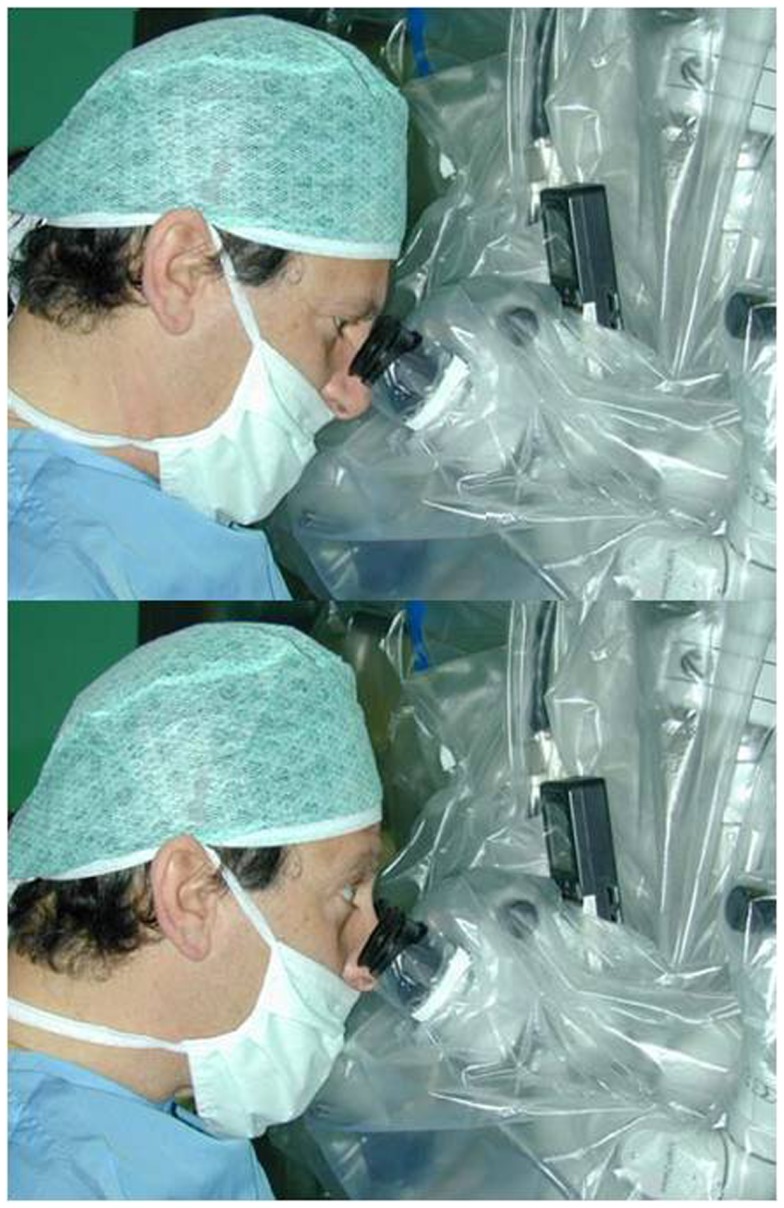
**The 7-inch high resolution LCD screen placed above the binocular head of the microscope to obtain a simultaneous vision of the endoscopic and microsurgical images by a simple gaze movement**.

The endoscopic system (Karl Storz GmbH and Co. KG, Tuttlingen, Germany, Hopkins Galzio Endoscope) consisted of two types of endoscopes: (1) Straight Forward Telescope with viewing angle 0°, outer diameter 2.7 mm, and working length 15 cm; (2) Hopkins-Forward Oblique Telescope with viewing angle 30°, diameter 2.7 mm, working length 15 cm, and with view direction at 6 and 12 o’clock position (Figure 1). The endoscope was fixed to the operative field by a mechanical holding system retractor arm, attached to the operative table. A xenon light source provided the adequate illumination. The endoscopic and microscopic images, in a “picture in picture” style, were viewed on a 7-inch high resolution LCD screen placed above the binocular head of the microscope, and on another 21-inch monitor (Karl Storz GmbH and CO. KG, Tuttlingen, Germany) (Figure 2).

**Figure 3:**
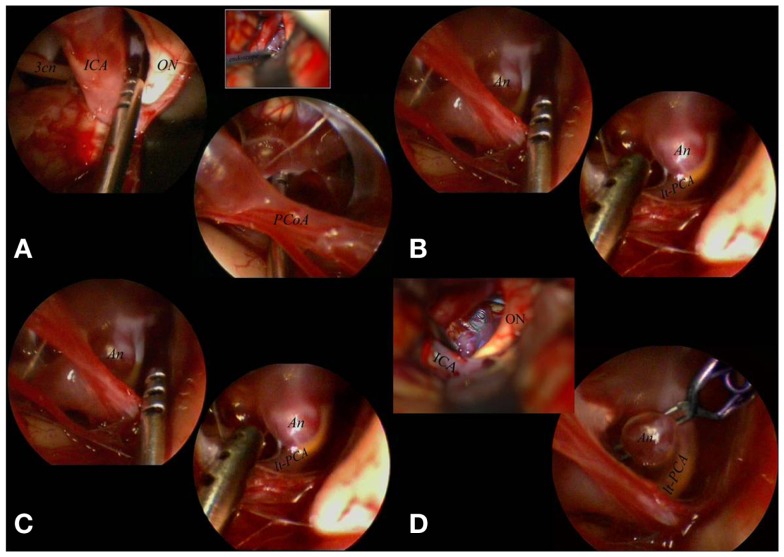
**The aneurysm was mobilized, under scopic control, with the microsucker passing beyond and behind the PCoA (A)**. With the sucker introduced below and in front of the PCoA the aneurysmal sac and the surrounding circulation were exposed **(B)**. The aneurysm was clipped passing with the clip applier in the corridor between ON and ICA, with the scope fixed to a mechanical holder to control the surgical maneuvers (comparing the microsurgical and the endoscopic views it is clear that the endoscope better visualized the distal portion of the clip **(C)**. The final endoscopic control allowed to confirm patency of the left PCA not clearly visible under the microscope **(D)**. 3cn, oculomotor nerve; An, aneurysm; ICA, internal carotid artery; lt, left; ON, optic nerve; PCA, posterior cerebral artery; PCoA, posterior communicating artery.

Standard tailored approaches, based on skull base surgery principles, were chosen. During the surgical procedures, the use of the endoscope included three steps: initial inspection, true operative time, and final inspection. During the first step, after microsurgical subarachnoid dissection and eventual washing away of blood clots to expose the aneurysmal region, the endoscope was introduced into the surgical field. The rigid endoscope 0° was introduced free-hand, under microscopic view, using the same microsurgical route. In this way, we were able to identify the lesional and perilesional anatomy. Whenever possible, a different corridor from the surgical one was implemented using the endoscope angled 30° with the most appropriate viewing direction. If the endoscope was considered to be useful in order to configurate the aneurysm shape, its localization, the direction of the aneurysmal sac, and its relation to the intracerebral vascular anatomy, it was fixed on the mechanical holding system. The adjustments of the endoscope position were maneuvered by the surgeon to obtain the optimal position and view, meanwhile the assistant fixed the holding system (10) (Figure 3).

**Figure 4:**
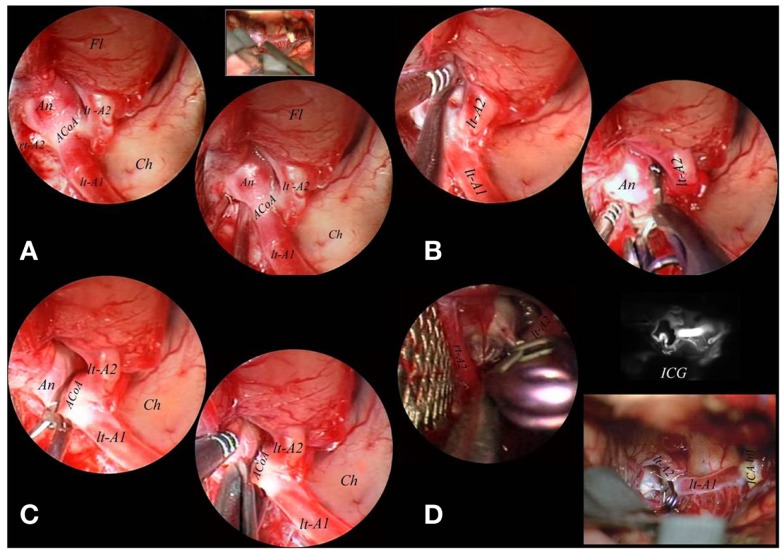
**The endoscope was fixed to a mechanical holder and the right A2 tract was freed from the medial border of the implant base under endoscopic control (A)**. Also the left A2 tract directly originating from the right A1 tract was dissected from the lateral border of the basal portion of the aneurysmal sac and a clip was applied under endoscopic control **(B)**. After definitive clipping was obtained, the sac was opened with microscissors to confirm complete exclusion **(C)**. A final inspection with the endoscope used free-hand evidentiated the complete exclusion of the lesion which was confirmed by intraoperative indocyanin green fluoroangiography **(D)**. A1, proximal segment of the anterior cerebral artery; A2, postcommunicating segment of the anterior cerebral artery; ACoA, anterior communicating artery; An, aneurysm; Ch, chiasma; Fl, frontal lobe; ICA bif, bifurcation of internal carotid artery; ICG, indocyanin green fluoroangiography; lt, left; M1, proximal segment of the middle cerebral artery; rt, right.

The following step (“true operative time step”) was performed under simultaneous microscopic-endoscopic control. It included the dissection of adherent vessels from the aneurysm, the exposure of the aneurysmal sac and neck, temporary clipping, placement and/or replacement of permanent clips, coarctation and/or resection of the fundus (Figure 4).

**Figure 5:**
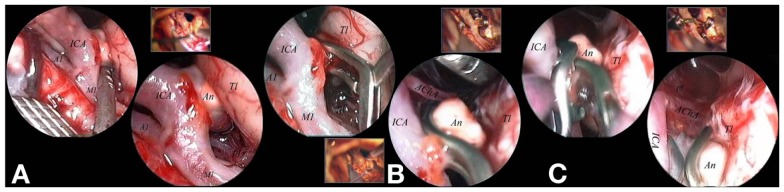
**The lesion appeared indoved into the temporal parenchyma (A)**. A straight forward 0° scope, used free-hand, allowed a clear vision of the high number of functional perforators surrounding the lesion, without any need to mobilize nor the nor the parental artery. Functional perforators were present both on the superior and of the inferior aspects of the aneurysmal implant base **(A)**. The scope was fixed and the aneurysm was clipped with a S-shaped clip applied under direct endoscopic control **(B)**. A second clip was applied in front of the first one to obtain the complete control of the implant base without endangering the anterior choroidal artery, which originated immediately anterior to the implant base itself **(C)**. A1, proximal segment of the anterior cerebral artery; AChA, anterior choroidal artery; An, aneurysm; ICA, internal carotid artery; M1, proximal segment of the middle cerebral artery; pf, perforator; Tl, temporal lobe.

The third step was the final inspection using the free-hand endoscope. The final inspection included the assessment of the complete exclusion of the aneurysm and, at the same time, the preserved patency of parent, branching and perforating vessels (Figure 5).

Moreover since inspection alone cannot reveal impeding ischemia, it was supported by complementary methods, such as intracranial ultrasound Doppler, intraoperative video fluoroangiography, and electrophysiological monitoring techniques.

## Results

Successful clipping was achieved in all cases. All patients underwent postoperative cerebral angiography. Follow-up ranged from 3 to 36 months. One hundred and fifty-six patients (90%) showed excellent or good recoveries (GOS of five or four). There were only two cases of mortality within 72 h of surgery: one for pulmonary embolism and the other death was due to acute myocardial infarction. The complications directly related to endoscopic procedures were rare. In our series we observed one transient oculomotor palsy and two small cerebral contusions, clinically silent, due to an inadvertent impingement to the nerve and to the cerebral parenchyma by the endoscope. There was no rupture of the aneurysm sac caused by the use of the endoscope.

In 147 aneurysms (71.4%), the endoscope provided additional information and its application did not prolong surgical time. In these cases the endoscope provided a better view of the regional anatomic features, assuring a clearer observation of parent, branching and perforating vessels, a better visualization of the neck and posterior wall of the aneurysm, confirming the complete exclusion of the aneurysmatic lesion, and/or excluding the inclusion of the parent artery in the clip.

In 35 cases (16.9%) the first temporary clipping was incorrectly performed and the clip was immediately removed. Clipping under pure endoscopic view was done in 42 cases (20.4%).

During the free-hand initial inspection, the 0° endoscope facilitated the observation of all regional anatomy, due to the illumination, the magnification, and the different perspective view of the surgical target. Proceeding with the 30° endoscope we gained information concerning the vascular network around the aneurysm.

In most cases of ICA-Opht artery aneurysms, arising from the anterior wall of the ICA, the endoscope did not provide further information. However, fixing the endoscope into the prechiasmatic cistern, the endoscope clarified the neck position relative to the distal ring. In cases of superior-hypophyseal location of the aneurysm the endoscope provided information regarding perforators and neck position. For all aneurysms of the posterior wall of the ICA (ICA-PcomA, ICA-AchorA), ICA bifurcation (Figure 5), and BA (Figure 4), Basilar tip, BA-superior cerebellar artery (BA-SCA), and proximal posterior cerebral artery (PCA) we used the 30° endoscope by clamping it to the surgical field and using endoscopic corridors complementary to microsurgical working channels. In these cases the simultaneous control of microscopic and endoscopic views facilitated the control of anatomical features, parent and perforating arteries, neck and posterior wall of aneurysms. Furthermore, the endoscope allowed the direct and/or the completeness of aneurysm clipping.

In MCA aneurysms, particularly for aneurysms located in the proximal tract (M1), the endoscope was hardly ever fixed to the surgical field as it was not able to add new information compared to free-hand initial inspection.

No utility was found for aneurysms of Midbasilar, VBJ, VA-PICA, and Distal artery (Pericallosal, Distal PcomA, Distal SCA, P1/P2, and P2/P3 tracts of the PCA). In these cases, the problem related to the endoscope use was the narrow working channel. For these locations, the simultaneous control of microscopic and endoscopic views and clipping was not possible because 30° angled endoscope proved to be useless. However, the 0° endoscope was used free-hand during the surgical initial inspection, especially during the first series of procedures.

## Discussion

### Instrumentations

We have been applying the endoscope for cerebral aneurysms since 1997, and in 2002 we began using specific endoscopic equipment (Karl Storz GmbH and Co. KG, Tuttlingen, Germany, Opkins Galzio Endoscope). Reviewing the literature, the rigid endoscopes, used during the assistance, had a range from 0° to 110° (8, 10–15). At the beginning of our experience we used rigid endoscopes with diameter of 4.0 mm and angle of view of 0°, 30°, and 45°. After a learning period with the first cases, the endoscope angled 45° was abandoned, using only rigid endoscopes with angle of view of 0° and 30°. In our experience, endoscope with a high degree could create confusion and disorientation in the mind of the surgeon.

During the endoscopic procedure the common problems of graduated endoscopes were the partial obstruction of the microscopic view caused by the camera, during the rotation of the scope, to obtain different visual perspectives (12, 13). To eliminate this defect, Tamaki used the body of the endoscope angled at 110°(13). However, in order to avoid this limitation, we have developed a specifically designed endoscope with a 45°-angled eyepiece, which allows different viewing directions, keeping camera head out of the operative view, avoiding that the scope itself obstructs the smooth flow of microsurgical maneuvers. Moreover, the instrumentation presents well-balanced ergonomic design which is comfortable and stable in all operating conditions, whether it is manually operated by the free-hand or used while mounted to a holder. The system also includes the use of a mechanical holders which allows a precise and atraumatic fixing into the operative field without endangering surgical maneuvers (Figures 3–5).

### Technique

An effective EAM for intracranial aneurysms requires the simultaneous vision by microscope and endoscope, but it may be difficult to adapt the 2-dimensional information of the endoscope and the 3-dimensional information of the microscope, which gives a wider sense of depth (16, 17). For these reasons, we have studied a system to observe microscopic images through the ocular system of the microscope and endoscopic images on a high resolution 7-inch LCD screen, placed above the ocular system, to obtain a simultaneous view by a simple gaze movement (18–20) (Figure 2). However, the learning curve of these procedures requires optimal knowledge of surgical anatomy and of endoscopic anatomy. Therefore, continuous practice is the key-point of this technique.

During surgery the endoscope must be considered a real surgical instrument, therefore it must be introduced in the operative field under microscopic view and clamped to the operative table to allow the bimanual manipulation. In our experience, the EAM was performed using endoscopic corridors complementary to microsurgical working channels: (1) the carotid-oculomotor space, laterally to the surgical corridor, for posterolateral wall carotid aneurysm (ICA-PcomA, ICA-AchorA); (2) the carotid-oculomotor space and the optico-carotid space, for BA aneurysms (Basilar tip, BA-SCA junction, proximal PCA) (12, 21–23). Through the carotid-oculomotor route, as endoscopic corridor, a microscopic manipulation could be safely done via the optico-carotid route, paying attention to the perforating arteries arising from ICA, PcomA, and AchorA, that may obstruct the optico-carotid space. In case of obstacle due to perforating vessels into the optico-carotid space, the endoscope may be used in the carotid-oculomotor space, laterally to the microsurgical corridor (Figure 4).

Finally, the illumination intensity of the endoscopic light source must be usually set at a very low output (never exceeding 10–20% of the maximum power), to avoid thermal injuries; this is possible because the endoscopic vision takes, in any case, advantage from the light beam of the microscope, which illuminates the operative field also in the deep sectors. In some instances, especially when the 0°-scope is used, to obtain clear endoscopic images it may be even necessary to switch off the endoscopic light source and/or to reduce the intensity of the microscopic lighting beam.

### Influencing factors and indications for EAM

For Taniguchi indications for EAM are unruptured aneurysms or aneurysms after SAH absorption (14). Zhao states that the endoscope is unsuitable for the treatment of ruptured aneurysms with SAH (Fisher grade III or higher), blood in the surgical field and brain swelling (15).

However, in our cases the presence of blood in the basal cisterns as well as the presence of hydrocephalus did not contraindicate endoscopy. The endoscope results useful with ruptured as well as with unruptured aneurysms and surgical timing is not influent. In any case, an adequate vision will be provided after cisternal opening and washing, performed during early microsurgical operative steps, with eventual opening of the lamina terminalis and/or performing an intraoperative ventricular drainage before the endoscopic introduction.

In our opinion, the most important factors contributing to the efficacy of endoscope-assisted microsurgical treatment of aneurysms are determined by their size and location. In general terms, very large and giant aneurysms gain less benefits from EAM than smaller ones in the same location, because the mass of the lesion can compromise insertion and fixation of the endoscope in the operative field. Moreover, these lesions are usually exposed through larger approaches, but the endoscope as an adjunctive optical device is helpful especially in minimally invasive “keyhole” approaches allowing various angles of view with less operating space.

However, the anatomical location of aneurysm is the most important factor to be considered in evaluating indications and thereafter efficacy and applicability of EAM. During surgery the endoscopic vision is possible only in preexisting anatomic cavitary spaces, and a clean operative field, with a sufficient surgical space, is the fundamental requisite to apply the endoscope (24).

With regards to anterior circulation (Table 2), for aneurysms localized in the ICA-anterior wall (ICA-Opht), EAM is little useful because these lesions are located in the superficial plane of the operative field and no perforators or arterial branches are hidden enough to be endangered during clipping. On the contrary, to treat aneurysms of ICA-posterior wall (ICA-PcomA, ICA-AchorA), the endoscope is extremely useful because these lesions are located in the depth of the operative field, hidden by carotid siphon and surrounded by numerous critical perforators. Moreover paracarotid cisterns offer enough space to introduce and fix the endoscope. In these cases, the technique can allow direct and correct clipping with minimal manipulation or retraction of the parental artery, avoiding damage to PcomA, AchorA, and many perforators (25, 26). For aneurysms of ICA bifurcation, EAM is extremely useful too, because these lesions are located in an arterial segment rich of perforators, often hidden by lesion itself, and that may be explored with minimal manipulation or retraction of the parental vessels, reducing the risk of intraoperative rupture and ischemic damage during clipping (Figure 4). Regarding aneurysms of AcomA/ACA complex, the endoscope is of limited benefit because these lesions are not located in a true cisternal space where the endoscope can be fixed without endanger surgical maneuvers. In these cases, the endoscopic procedure may be however useful using free-hand scopes to explore anatomy before and after clipping (Figure 5). Finally, EAM is not effective for lesions of MCA, because these aneurysms are superficially located in the operative field, they don’t lie in a true cisternal space, and they may be normally manipulated in order to expose covered arterial branches. No utility has been found for pericallosal aneurysms.

**Table 2:** **Anatomical localizations that gain advantages or not from the endoscopic procedures**.

Anatomical localization	Useful	Not useful	Limited useful
MCA		X	
ICA-anterior wall (ICA/Opht)			X
ICA-posterior wall (ICA/AchorA, ICA/PcomA)	X		
ICA bifurcation	X		
ACA/AcomA			X
BA	X		
MA			X
VBJ			X
VA			X
Distal arteries		X	

*MCA, middle cerebral artery; ICA, internal carotid artery; Opht, ophthalmic; AchorA, anterior choroidal artery; PcomA, posterior communicating artery; ACA, anterior cerebral artery; AcomA, anterior communicating artery; BA, basilar artery; MA, midbasilar artery; VBJ, vertebral-basilar junction; VA, vertebral artery*.

As regards as posterior circulation (Table 2), we observed that EAM is extremely useful for aneurysms of the BA (Basilar tip, BA-SCA, and proximal PCA), first of all because these lesions are deeply located below and behind critical neurovascular structures with an arterial segment rich of vital perforators (25, 27) (Figure 3). Moreover the direct clipping is possible because these aneurysms lie in large cisternal spaces enough to fix the scope into the operative field without endanger surgical maneuvers. For Midbasilar aneurysms, EAM is of little value because these lesions are embedded in very limited subarachnoid spaces, where the endoscope cannot be fixed or easily introduced. Moreover these lesions are exposed through skull base approaches and appear in the immediate surface of the operative field. The same principle is effective for lesions of Vertebro-basilar junction and Vertebral/PICA region. In these two last cases, EAM may be helpful using free-hand scopes to visualize perilesional anatomy (perforators) before and after clipping. No utility has been found for the aneurysms of Distal PcomA, Distal SCA, P1/P2, P2/P3, although further cases should be treated to definitely validate the procedure for these locations.

## Conclusion

The endoscope is not useful for all locations of the aneurysms. EAM is especially useful in the treatment of deeply located cerebral aneurysms, located in arterial segments rich of perforators, and in anatomical regions where space is enough to introduce and move the endoscope without endangering microsurgical maneuvers. The endoscopic procedure doesn’t require special surgical instruments (most of normally used microsurgical instruments result effective) and the advantages are particularly evident using specific scopes and holders, after adequate surgical training. For these reasons, we recommend keeping the endoscope ready in the operating room in all cases of surgery of aneurysms. In this way, the neurosurgeon can become aware of the risks of insertion of the endoscope into the surgical field and can increase his learning curve. In fact, the use of the endoscope requires solid bases of surgical and endoscopic anatomy to reach the following goals in the treatment of intracranial aneurysms: control of the regional anatomy, clipping under simultaneous microscopic and endoscopic views, and confirmation of the complete exclusion of the aneurysmatic sac.

## Conflict of Interest Statement

The authors declare that the research was conducted in the absence of any commercial or financial relationships that could be construed as a potential conflict of interest.
